# Evaluation of the Glucocorticoid-Sparing Effect of Adding Methotrexate in Polymyalgia Rheumatica: A Single-Center Retrospective Study

**DOI:** 10.7759/cureus.89107

**Published:** 2025-07-31

**Authors:** Junya Kitai, Hirofumi Miyake, Ryuichi M Sada, Yukio Tsugihashi, Kazuhiro Hatta

**Affiliations:** 1 Department of General Internal Medicine, Tenri Hospital, Nara, JPN; 2 Inflammation and Immunology, Graduate School of Medical Science, Kyoto Prefectural University of Medicine, Kyoto, JPN; 3 Department of Infection Control, Graduate School of Medicine, The University of Osaka, Osaka, JPN; 4 Department of Transformative Protection to Infectious Disease, Graduate School of Medicine, The University of Osaka, Osaka, JPN; 5 Medical Home Care Centre, Tenri Hospital Shirakawa Branch, Nara, JPN

**Keywords:** flare, glucocorticoid resistance, glucocorticoid-sparing effect, methotrexate, polymyalgia rheumatica

## Abstract

Objective

This study assessed whether the addition of methotrexate (MTX) to glucocorticoid therapy reduces overall glucocorticoid usage in patients with polymyalgia rheumatica (PMR), focusing particularly on those exhibiting glucocorticoid-resistant disease.

Methods

This retrospective study included 82 patients with PMR treated with glucocorticoids. After applying matching weights, outcomes were compared between weighted populations of 18 patients receiving MTX and 18 receiving glucocorticoid monotherapy. A subgroup analysis was conducted among the 38 patients classified as glucocorticoid-resistant, comparing weighted populations of eight in the MTX group and nine in the non-MTX group after the application of matching weights.

Results

In the primary analysis (overall cohort), the cumulative glucocorticoid dose at 104 weeks was 4,263 mg in the MTX group and 4,418 mg in the non-MTX group, showing no significant reduction effect from MTX (mean difference, -155 mg; 95% confidence interval (CI), -1003 to 693; P = 0.717). The glucocorticoid discontinuation rate also showed no significant difference between the groups (38.2% vs. 27.8%; risk ratio (RR), 1.37; 95% CI, 0.61 to 3.1; P = 0.442). By contrast, in the subgroup analysis of glucocorticoid-resistant patients, the cumulative glucocorticoid dose in the MTX group was 4,298 mg, which was significantly lower than the 5,618 mg in the non-MTX group, with a mean difference of -1320 mg (95% CI, -2539 to -102; P = 0.035). The glucocorticoid discontinuation rate was also significantly higher in the MTX group than in the non-MTX group (45.5% vs. 4.1%; RR, 11; 95% CI, 2.1 to 57.9; P = 0.0059).

Conclusions

Although adjunctive MTX therapy did not significantly reduce glucocorticoid exposure in the overall PMR population, our findings suggest a substantial benefit for patients with glucocorticoid-resistant PMR, markedly reducing cumulative glucocorticoid exposure and increasing the rate of discontinuation. These results highlight the critical importance of patient selection when considering MTX for PMR.

## Introduction

Polymyalgia rheumatica (PMR) is a common inflammatory condition primarily affecting middle-aged and older individuals, characterized by proximal limb pain, morning stiffness, and elevated inflammatory markers such as C-reactive protein (CRP) and erythrocyte sedimentation rate (ESR) [1]. Glucocorticoids remain the cornerstone of PMR treatment due to their rapid efficacy and their ability to control frequent relapses; however, long-term use is frequently compromised by a high incidence of adverse events [2,3]. This underscores the need for alternative strategies that can reduce glucocorticoid exposure.

Several immunosuppressive agents have been evaluated as adjunctive therapies in PMR. Notably, MTX has long been considered a treatment option for PMR, with some studies demonstrating its efficacy, supported by extensive clinical experience, substantial research background, and cost-effectiveness [4-10]. In 2015, the European Alliance of Associations for Rheumatology (EULAR) and the American College of Rheumatology (ACR) conditionally recommended early MTX introduction alongside glucocorticoids for patients at high risk of relapse or likely to require prolonged therapy, while also suggesting its use during follow-up for those who relapse or respond insufficiently to glucocorticoids [4]. Previous reports have not reached a consistent conclusion regarding the benefit of adding MTX to PMR treatment across all patients [5-10]. This variability may be attributable to differences in patient backgrounds; specifically, although some PMR patients achieve rapid remission with glucocorticoid monotherapy, others exhibit resistance, which can obscure the potential benefits of MTX add-on therapy. It is conceivable that limiting the analysis to glucocorticoid‐resistant patients could clearly reveal the effectiveness of adjunctive MTX. Nonetheless, no study has specifically evaluated the effect of MTX add-on therapy by stratifying PMR patients according to their treatment responsiveness.

Therefore, the primary aim of this study was to test the hypothesis that MTX add-on therapy reduces glucocorticoid dependence in patients with PMR. To this end, we compared the cumulative glucocorticoid dose and discontinuation rates in the overall cohort. Furthermore, we also aimed to investigate whether this effect was more evident in a subgroup of glucocorticoid-resistant PMR patients.

## Materials and methods

Study design and setting

This retrospective study was performed in the general internal medicine outpatient clinic of Tenri Hospital, a 715-bed regional tertiary care teaching hospital in Japan, from September 1, 2020, to December 31, 2022. Tenri Hospital provides medical care for patients with autoimmune inflammatory rheumatic diseases in the General Internal Medicine Department, with the staff comprising 17 physicians, including three rheumatology specialists (as of September 1, 2020).

The study protocol was approved by the Ethics Review Board of Tenri Hospital (approval number: 1383), and the study was performed following the ethical standards laid down in the appropriate version of the WMA Declaration of Helsinki-Ethical Principles for Medical Research Involving Human Subjects. The Institutional Review Board of Tenri Hospital waived the requirement for informed consent due to the retrospective nature of the study, as all participants were given an opportunity to opt out. The present study followed the Strengthening of the Reporting of Observational Studies in Epidemiology statement [11].

Participants

All consecutive patients with PMR who visited the general internal medicine outpatient department of Tenri Hospital between September 1, 2020, and November 30, 2020, were enrolled in the present study. The inclusion criteria were a clinical diagnosis of PMR by the attending physician and treatment with a glucocorticoid. The exclusion criteria were 1) development of PMR before 2006, 2) treatment initiation at other hospitals, 3) termination of follow-up during the observation period, 4) receipt of disease-modifying anti-rheumatic drugs (DMARDs) or immunosuppressive drugs other than MTX during the observation period, 5) concurrent giant cell arteritis (GCA), 6) receipt of cytotoxic chemotherapy for malignancy during the observation period, 7) positive anti-citrullinated protein/peptide antibody (ACPA), 8) fulfillment of the 2010 ACR/EULAR classification criteria for rheumatoid arthritis (RA) or a diagnosis of other inflammatory arthritides (e.g., psoriatic arthritis), and 9) absence of new-onset PMR. The first three exclusion criteria were chosen because of the difficulty in accurately calculating the cumulative glucocorticoid dosage in such patients. The fourth exclusion criterion was chosen because drugs other than MTX are not recommended by the guidelines, and there are few studies showing efficacy [4]. The fifth exclusion criterion was chosen because of the different intensity of treatment required. The sixth exclusion criterion was chosen because cytotoxic chemotherapy during the observation period may cause significant immunosuppression, potentially influencing treatment outcomes. The seventh and eighth exclusion criteria were chosen to ensure a clear PMR cohort by excluding patients with a high possibility of, or a confirmed diagnosis of, other inflammatory arthritides such as RA. The ninth exclusion criterion was chosen because cumulative glucocorticoid administration could affect treatment efficacy. 

Variables and data collection

Data pertaining to the following variables were collected from patients’ medical records: variables at the last evaluation before the initiation of glucocorticoids for PMR: age, sex, CRP level (mg/dL), ESR (mm/h), matrix metalloproteinase-3 level (MMP-3) (ng/mL), estimated glomerular filtration rate (eGFR) (mL/min/1.73 m^2^), aspartate aminotransferase (AST) level (IU/L), alanine aminotransferase (ALT) level (IU/L), alkaline phosphatase (ALP) level (IU/L), rheumatoid factor (RF) level (IU/mL), ACPA level (U/mL), and initial dose of glucocorticoids (mg/day).

In addition, the presence of comorbidities such as hypertension, diabetes mellitus, dyslipidemia, cardiovascular disease, cerebrovascular disease, osteoporosis, and ocular complications (including cataract and glaucoma) was recorded at baseline.

Variables of patients receiving MTX: number of days from glucocorticoid initiation to MTX initiation, maximum dose of MTX administered during the observation period (mg/week), glucocorticoid dose at the time of MTX initiation (mg/day), cumulative glucocorticoid dose before MTX initiation (mg), CRP level (mg/dL) and ESR (mm/h) at MTX initiation, and MTX-related adverse events.

Cumulative glucocorticoid dosage over 104 weeks from the time of glucocorticoid initiation, glucocorticoid discontinuation rate at 104 weeks after glucocorticoid treatment initiation, and number of flares at 104 weeks after glucocorticoid treatment initiation. Flare was defined as a recurrence of symptoms or an elevated inflammatory response (ESR/CRP level) requiring an increased glucocorticoid dosage [12]. 

Exposures

Patients with a history of MTX treatment during the observation period were categorized in the MTX group, whereas those without a history of MTX treatment during the observation period were categorized in the non-MTX group. All MTX prescriptions were administered within the scope of Japan's national health insurance, typically as a glucocorticoid-sparing agent.

Candidate risk factors 

The following baseline factors have been identified as potential risk factors for cumulative glucocorticoid dosage, glucocorticoid discontinuation rate, and number of flares: age, sex, initial glucocorticoid dose, MTX use (MTX use within 104 weeks of glucocorticoid initiation), and values of ESR, CRP, MMP-3, eGFR, AST, ALT, ALP, RF, and ACPA.

The reasons for choosing these factors were as follows: age is associated with the continuation of glucocorticoid treatment in older patients [13]; women are more likely to experience relapses, higher cumulative glucocorticoid dosages, longer treatment periods, and lower discontinuation rates after two years [14-18]; the CRP level is associated with relapses and the duration of glucocorticoid treatment [14,15,18,19]; ESR is related to relapses, the two-year discontinuation rate, and treatment duration [14,16,18,20,21]; the ALP level is associated with relapses [22]; the initial glucocorticoid dosage is associated with discontinuation rates [13]; and the MMP-3 level is related to disease activity [23]. In addition, AST and ALT levels increase in patients with PMR [24]. eGFR, AST, and ALT values were chosen owing to their relevance in determining the feasibility of MTX administration.

Outcomes

The primary outcomes were the cumulative glucocorticoid dosage over 104 weeks from the time of glucocorticoid initiation and the glucocorticoid discontinuation rate at 104 weeks after the initiation of glucocorticoid therapy. The secondary outcomes were the number of flares, defined as the recurrence of symptoms or an increase in inflammatory markers requiring an increase in the glucocorticoid dosage (counted as one flare if the dosage increase occurred consecutively two or more times), and the frequency and types of MTX-related adverse events. MTX-related adverse events included all symptoms and laboratory abnormalities that required dose reduction or discontinuation during treatment.

Statistical analyses

Patient characteristics are described using the median and interquartile range for continuous variables and number and percentage (%) for categorical variables. The Wilcoxon rank-sum test was used to compare continuous variables between the two groups, whereas the Pearson chi-squared test was used to compare categorical variables. For descriptive purposes, analyses of variables with missing data were conducted on the available cases. All covariates used in the propensity score model (age, sex, CRP level, eGFR, and initial glucocorticoid dose) were complete, with no missing values.

We used matching weights to adjust for baseline confounding. Propensity scores were estimated using a logistic regression model with the following baseline covariates, selected for their clinical association with the treatment and outcomes: age, sex, CRP level, eGFR, and initial glucocorticoid dose. Notably, CRP was chosen over ESR as the marker of disease severity due to missing ESR data in seven cases.

After applying the matching weights, the outcomes were compared between the MTX and non-MTX groups using statistical models appropriate for each outcome's characteristics. The mean difference in the cumulative glucocorticoid dose at 104 weeks was calculated using a generalized linear model. The risk ratio (RR) for glucocorticoid discontinuation at 104 weeks was calculated using a log-binomial regression model adjusted for overdispersion. The incidence rate ratio (IRR) for the number of flares at 104 weeks was calculated using a Poisson regression model adjusted for overdispersion.

Furthermore, subgroup analyses were conducted in patients with glucocorticoid-resistant PMR, defined in this study as those who required an increase in the glucocorticoid dosage before tapering prednisolone to less than 5 mg/day, due to PMR-related symptoms and/or elevated inflammatory markers such as ESR or CRP [25,26].

The statistical significance level was set at 0.05. All analyses were performed using the R software program (version 4.4.2; R Foundation for Statistical Computing, Vienna, Austria) within RStudio (version 2024.12.0+467).

## Results

Study flow

From the 137 patients meeting the inclusion criteria, we excluded two patients with onset before 2006, 23 patients with treatment initiation at other hospitals, one patient with follow-up termination during the observation period, 16 patients who received DMARDs or immunosuppressants besides MTX during the observation period, one patient who received cytotoxic chemotherapy for malignancy, seven patients with concurrent GCA, four patients with ACPA positivity, and one patient who did not have new-onset PMR. No patients were excluded for meeting the 2010 ACR/EULAR classification criteria for RA or having a diagnosis of other inflammatory arthritides. As a result of this exclusion process, 82 patients treated with glucocorticoids were included in the analysis. In addition, 38 patients with glucocorticoid-resistant PMR were included in the subgroup analyses.

Patient characteristics

The patient characteristics and outcomes before and after applying matching weights are summarized in Table 1.

**Table 1:** Baseline characteristics of glucocorticoid-treated polymyalgia rheumatica patients with or without MTX before and after matching weights. The propensity score was calculated using the following variables: age, sex, CRP, eGFR, and initial glucocorticoid dose. Abbreviations: MTX, methotrexate; SMD, standardized mean difference; IQR, interquartile range; ESR, erythrocyte sedimentation rate; CRP, C–reactive protein; eGFR, estimated glomerular filtration rate; AST, aspartate aminotransferase; ALT, alanine aminotransferase; ALP, alkaline phosphatase; RF, rheumatoid factor; MMP–3, matrix metalloproteinase–3; NA, not applicable

	MTX group (before weighting) n = 20	Non-MTX group (before weighting) n = 62	p-value	MTX group (after weighting) n = 18	Non–MTX group (after weighting) n = 18	p-value	SMD
Age, years, median (IQR)	73 (66, 79)	75 (69, 79)	0.38	73 (66, 79)	74 (68, 77)	0.8	0.083
Male, sex, n (%)	12 (60)	23 (37.1)	0.12	10.3 (56.4)	10.5 (57.3)	0.95	0.019
Height, cm, median (IQR)	159 (153, 167)	156 (152, 164)	0.4	160 (153, 167)	157 (152, 163)	0.3	0.231
Weight, kg, median (IQR)	54 (50, 65)	53 (46, 58)	0.2	54 (49, 63)	53 (47, 59)	0.6	0.157
Hypertension, n (%)	7 (35)	28 (45.2)	0.59	7 (38.2)	9.1 (49.8)	0.42	0.236
Diabetes mellitus, n (%)	2 (10)	9 (14.5)	0.89	2 (10.9)	2.2 (11.8)	0.92	0.029
Dyslipidemia, n (%)	6 (30)	14 (22.6)	0.71	5.7 (30.9)	3.8 (20.9)	0.42	0.229
Cardiovascular disease, n (%)	1 (5)	9 (14.5)	0.46	1 (5.5)	2.9 (15.7)	0.29	0.337
Cerebrovascular disease, n (%)	2 (10)	7 (11.3)	1	1.7 (9.1)	2.4 (13.2)	0.66	0.132
Osteoporosis, n (%)	2 (10)	2 (3.2)	0.53	2.0 (10.9)	0.5 (2.9)	0.19	0.32
Ocular complications, n (%)	0 (0)	3 (4.8)	0.75	0 (0)	1.2 (6.5)	0.22	0.373
ESR, mm/h, median (IQR)	84 (68, 99)	75 (56, 91)	0.14	83 (68, 99)	84 (59, 100)	0.6	0.324
CRP, mg/dL, median (IQR)	8.2 (6, 10.4)	5.49 (3.02, 8.68)	0.01	7.82 (5.91, 10.15)	8.01 (5.37, 13.53)	0.87	0.088
eGFR, mL/min/1.73m^2^, median (IQR)	83.1 (71.8, 92)	74 (60.8, 90.8)	0.28	82.4 (70.1, 91.1)	82.5 (65.1, 97.4)	1	0.025
AST, IU/L, median (IQR)	19 (17, 29)	18 (15, 23)	0.22	18 (16, 29)	20 (16, 26)	0.76	0.127
ALT, IU/L, median (IQR)	18 (11, 30)	13 (9, 20)	0.08	17 (10, 25)	15 (10, 25)	0.64	0.194
ALP, U/L, median (IQR)	283 (241, 347)	261 (232, 323)	0.47	276 (226, 305)	273 (225, 334)	0.95	0.035
RF positivity, n (%)	1 (5.3)	2 (3.4)	1	1.0 (5.8)	0.2 (1.2)	0.15	0.254
MMP-3, ng/mL, median (IQR)	212 (120, 416)	182 (119, 322)	0.55	163 (112, 322)	227 (131, 323)	0.66	0.051
Starting dose of glucocorticoids, mg/day, median (IQR)	15 (15, 15)	15 (15, 15)	0.46	15 (15, 15)	15 (15, 15)	0.53	0.014
Days until MTX initiation, days, median (IQR)	151 (71, 410)	NA (NA, NA)		154 (110, 399)	NA (NA, NA)		NA
Maximum dose of MTX, mg/week, median (IQR)	6 (4, 8)	NA (NA, NA)		6 (4, 8)	NA (NA, NA)		NA

Before weighting, the median ages were 73 and 75 years in the MTX and non-MTX groups, respectively; the proportions of men were 60% and 37.1% in the MTX and non-MTX groups, respectively; and CRP levels were significantly higher in the MTX group than in the non-MTX group. After weighting, the SMDs for the baseline variables-age, sex, CRP level, eGFR, and initial glucocorticoid dose-were all below the conventional threshold of 0.1, indicating that the covariates were well-balanced in the weighted population.

Similarly, the patient characteristics and outcomes before and after weighting in the subgroup analysis of patients with glucocorticoid-resistant PMR are shown in Table 2.

**Table 2:** Baseline characteristics of glucocorticoid-resistant polymyalgia rheumatica patients with or without methotrexate before and after matching weights The propensity score was calculated using the following variables: age, sex, CRP, eGFR, and initial glucocorticoid dose. Abbreviations; MTX, methotrexate; SMD, standardized mean difference; IQR, interquartile range; ESR, erythrocyte sedimentation rate; CRP, C–reactive protein; eGFR, estimated glomerular filtration rate; AST, aspartate aminotransferase; ALT, alanine aminotransferase; ALP, alkaline phosphatase; RF, rheumatoid factor; MMP–3, matrix metalloproteinase–3; NA, not applicable

	MTX group (before weighting) n = 13	Non-MTX group (before weighting) n = 25	p-value	MTX group (after weighting) n = 9	Non-MTX group (after weighting) n = 8	p-value	SMD
Age, years, median (IQR)	73 (66, 80)	75 (69, 80)	0.32	73 (67, 79)	75 (69, 78)	0.93	0.033
Male sex, n (%)	10 (76.9)	10 (40)	0.069	6.3 (71.6)	5.7 (67.9)	0.84	0.079
Height, cm, median (IQR)	154 (152, 167)	156 (150, 161)	0.73	154 (153, 167)	159 (148, 162)	0.66	0.113
Weight, kg, median (IQR)	52 (49, 57)	53 (45, 57)	0.62	50 (46, 53)	53 (45, 59)	0.49	0.321
Hypertension, n (%)	2 (15.4)	9 (36)	0.34	1.6 (18.1)	1.9 (22.8)	0.77	0.118
Diabetes mellitus, n (%)	0 (0)	4 (16)	0.33	0 (0)	0.7 (8.1)	0.12	0.421
Dyslipidemia, n (%)	3 (23.1)	8 (32)	0.84	1.9 (22.1)	1.9 (22.7)	1	0.016
Cardiovascular disease, n (%)	0 (0)	4 (16)	0.33	0 (0)	1.8 (21.1)	0.081	0.732
Cerebrovascular disease, n (%)	2 (15.4)	3 (12)	1	0.8 (9.5)	0.7 (8.8)	0.95	0.023
Osteoporosis, n (%)	1 (7.7)	1 (4)	1	1 (11.4)	0.1 (0.9)	0.03	0.446
Ocular complications, n (%)	0 (0)	0	NA	0 (0)	0 (0)	NA	<0.001
ESR, mm/h, median (IQR)	75 (61, 87)	71 (51, 90)	0.72	72 (60, 81)	75 (52, 100)	0.63	0.09
CRP, mg/dL, median (IQR)	8.34 (5.85, 10.2)	5.5 (2.55, 8.96)	0.056	6.89 (5.54, 9.25)	6.92 (2.77, 13.37)	0.97	0.065
eGFR, mL/min/1.73m^2^, median (IQR)	82.1 (70.2, 88.5)	71.7 (59.2, 90.8)	0.38	77.3 (66, 85.2)	71.3 (64.1, 90.8)	0.97	0.067
AST, IU/L, median (IQR)	18 (16, 28)	19 (16, 25)	0.64	18 (15, 24)	20 (16, 25)	0.78	0.221
ALT, IU/L, median (IQR)	19 (11, 67)	15 (10, 20)	0.18	17 (10, 22)	17 (14, 23)	0.95	0.262
ALP, U/L, median (IQR)	291 (249, 418)	297 (250, 341)	0.9	283 (245, 295)	300 (245, 366)	0.44	0.019
RF positivity, n (%)	0 (0)	0 (0)	NA	0 (0)	0 (0)	NA	<0.001
MMP-3, ng/mL, median (IQR)	194 (106, 272)	154 (107, 241)	0.59	120 (97, 247)	156 (119, 234)	0.64	0.062
Starting dose of glucocorticoids, mg/day, median (IQR)	15 (15, 15)	15 (15, 15)	0.61	15 (15, 15)	15 (15, 20)	0.6	0.023
Days until MTX initiation, days, median (IQR)	148 (81, 399)	NA (NA, NA)		399 (110, 399)	NA (NA, NA)		NA
Maximum dose of MTX, mg/week, median (IQR)	6 (4, 6)	NA (NA, NA)		4 (4, 6)	NA (NA, NA)		NA

Before weighting, the median ages were 73 and 75 years in the MTX and non-MTX groups, respectively; the proportions of men tended to be higher in the MTX group (76.9%) than in the non-MTX group (40%); and CRP levels also tended to be higher in the MTX group than in the non-MTX group. After weighting, the SMDs for the covariates used to estimate the propensity score (age, sex, CRP level, eGFR, and initial glucocorticoid dose) were also well-balanced, with all values below 0.1.

Outcomes

The outcomes after weighting are presented in Table 3 (overall cohort) and Table 4 (glucocorticoid-resistant subgroup).

**Table 3:** Outcomes over 104 weeks in polymyalgia rheumatica patients with or without methotrexate: Comparison after matching weights The methotrexate non-use group served as the reference for all analyses. Abbreviations: CI, confidence interval; MTX, methotrexate

	MTX group n = 18	Non-MTX group n = 18	Effect measure	Estimate		p-value
Cumulative glucocorticoid dose of 104 weeks, mg, (95%CI)	4263 (3696, 4830)	4418 (3805, 5031)	Mean difference		-155 (-1003, 693)	0.717
Glucocorticoid discontinuation rate at 104 weeks, % (95%CI)	38.2 (16.1, 60.2)	27.8 (12.4, 43.2)	Risk ratio	1.37 (0.61, 3.1)		0.442
Number of flares at 104 weeks, median (95%CI)	1.4 (1, 1.9)	1.2 (0.8, 1.6)	Incidence rate ratio	1.21 (0.76, 1.92)		0.424

**Table 4:** Outcomes over 104 weeks in glucocorticoid-resistant polymyalgia rheumatica patients with or without methotrexate: Comparison after matching weights The methotrexate non-use group served as the reference for all analyses. Abbreviations: CI, confidence interval; MTX, methotrexate

	MTX group n = 8	Non-MTX group n = 9	Effect measure	Estimate	p-value
Cumulative glucocorticoid dose of 104 weeks, mg, (95%CI)	4298 (3543, 5052)	5618 (4714, 6522)	Mean difference	-1320 (-2539, -102)	0.035
Glucocorticoid discontinuation rate at 104 weeks, % (95%CI)	45.5 (14.7, 76.2)	4.1 (1.9, 10.2)	Risk ratio	11 (2.1, 57.9)	0.0059
Number of flares at 104 weeks, median (95%CI)	1.8 (1.3, 2.2)	2.1 (1.5, 2.7)	Incidence rate ratio	0.83 (0.55, 1.23)	0.34

In the overall cohort of patients with PMR, the mean difference in the cumulative glucocorticoid dose over 104 weeks was -155 mg (95% confidence interval (CI), -1003 to 693; p = 0.717). The RR for glucocorticoid discontinuation was 1.37 (95% CI, 0.61 to 3.1; p = 0.442). Regarding the number of flares, the IRR was 1.21 (95% CI, 0.76 to 1.92; p = 0.424), indicating no statistically significant difference (Table 3).

In the glucocorticoid-resistant subgroup, the mean cumulative glucocorticoid dose was lower in the MTX group by an estimated -1320 mg (95% CI, -2539 to -102; p = 0.035). The rate of glucocorticoid discontinuation at 104 weeks was higher in the MTX group (45.5% vs. 4.1%), with an estimated RR of 11 (95% CI, 2.1 to 57.9; p = 0.0059). Conversely, there was no significant difference in the rate of flares, with an IRR of 0.83 (95% CI, 0.55 to 1.23; p = 0.34) (Table 4).

The detailed clinical information for the MTX group is shown in Table 5.

**Table 5:** Detailed clinical information of patients in the methotrexate group Abbreviations: MTX, methotrexate; CRP, C-reactive protein; ESR, erythrocyte sedimentation rate; NI, no information

Case	Age (years)	Sex	Days until MTX initiation (days)	Maximum dose of MTX (mg/week)	Glucocorticoid dose just before MTX initiation (mg/day)	Cumulative glucocorticoid dose until MTX initiation (mg)	CRP at the start of MTX (mg/dL)	ESR at the start of MTX (mm/h)	Change in glucocorticoid dose during the observation period	MTX-related adverse events
1	60	Male	154	8	8	1706.5	1.83	16	Increased	None
2	81	Male	110	6	9	1360.5	1.2	25	Increased	None
3	61	Male	622	6	5	6083	0.16	2	Increased	None
4	66	Female	18	6	15	285	2.03	32	Increased	None
5	68	Male	126	4	7.5	1572.5	1.54	17	Increased	None
6	78	Male	32	6	15	495	1.04	NI	Increased	None
7	85	Female	28	6	15	435	1.34	13	Increased	Gastrointestinal symptom
8	85	Female	148	4	10	2390	0.2	NI	Increased	None
9	87	Female	399	4	12.5	495	0.1	4	Increased	None
10	73	Female	441	4	8	4003	0.5	40	Increased	None
11	73	Male	691	4	9	6576.5	0	8	Increased	None
12	73	Male	25	8	15	390	2.34	NI	Increased	None
13	74	Male	81	4	11	1203.5	0	8	Increased	None
14	44	Male	231	8	6	2793	0.42	2	Increased	None
15	69	Male	553	4	12.5	3247	0	6	Increased	None
16	81	Female	140	10	10	1662.5	0.21	27	Increased	None
17	71	Female	168	8	7	1722	0.08	18	Increased	None
18	77	Male	469	8	3	2770	1.03	21	Not increased	None
19	66	Female	161	10	9	1743	0.32	NI	Not increased	None
20	60	Male	42	6	12.5	630	0.68	29	Not increased	Liver injury

The median days from glucocorticoid to MTX initiation were 151, and the median maximum dose of MTX was 6 mg/week in the MTX group. The incidence of MTX-related adverse events was 2/20 (10%), with gastrointestinal symptoms (CTCAE grade 1) and liver injury (grade 2), both of which were manageable by reducing the dosage of MTX from 6 mg/week to 4 mg/week, resulting in improvement of the adverse events. No patient required discontinuation of MTX during the observation period.

## Discussion

The main findings of the present study, based on an analysis with matching weights, are as follows: 1) In the overall cohort of patients with PMR, the addition of MTX did not result in a significant reduction in glucocorticoid exposure. 2) However, in the subgroup of patients with glucocorticoid-resistant PMR, adjunctive MTX therapy was associated with a statistically significant reduction in the cumulative glucocorticoid dose and a significantly higher rate of glucocorticoid discontinuation. 3) MTX-related adverse events were observed in 10% of MTX users. This study is one of the few to specifically assess the effect of MTX in a real-world, glucocorticoid-resistant PMR cohort - a population that has been largely understudied.

Previous reports have not reached a consistent conclusion regarding the benefit of adding MTX to PMR treatment [4-10]. Our finding of no significant effect in the overall PMR cohort aligns with this ambiguity. However, the key finding of this study is the starkly different outcome observed in the glucocorticoid-resistant subgroup. By isolating this population, we were able to demonstrate that MTX provides a clinically meaningful and statistically significant benefit, reducing the cumulative glucocorticoid dose by approximately 1,320 mg over two years and increasing the risk ratio of discontinuation by over 11-fold. This highlights the critical importance of patient selection. The lack of a clear benefit in the overall cohort is likely due to the inclusion of patients who respond well to glucocorticoids alone, which masks the effect of adjunctive therapy. Our findings suggest that targeting MTX therapy to patients who exhibit resistance to glucocorticoids is an effective strategy.

The median maximum MTX dose in our study (6 mg/week) was notably lower than that used in many previous randomized controlled trials, which often utilized 7.5 mg/week or higher [4-10]. This difference likely reflects real-world clinical practice tailored to our patient population, which was characterized by advanced age (median 73 years) and a relatively low median body weight (54 kg). While this cautious approach likely contributed to the favorable safety profile observed, its impact on efficacy warrants careful interpretation. This lower dosage may explain why a statistically significant effect was not observed in the overall PMR population. However, our results demonstrate that in the treatment-resistant subgroup, where the potential for benefit is highest, even this modest dosage was sufficient to produce a statistically significant effect. It remains possible that a higher dose could have yielded an even greater benefit.

The limitations of this study include the following: 1) The small sample size, particularly in the subgroup analysis, limited the statistical power and contributed to the wide CIs observed for some outcomes. This imprecision for some key estimates, such as the RR for glucocorticoid discontinuation, means the results should be interpreted with caution and require confirmation in larger studies. 2) Baseline CRP levels were higher in the MTX group, suggesting that physicians may have preferentially prescribed MTX to patients with higher disease activity (confounding by indication). Although we used matching weights to adjust for this and other measured baseline differences, the potential for unmeasured confounding variables to influence the results remains a limitation. 3) As this study is a single-center retrospective investigation, its limitations include missing data and the possibility that treatment guidelines, including MTX dosing criteria, may differ from those at other hospitals. Due to the unavailability of crucial information required for PMR classification (e.g., the duration of stiffness), patient selection was based solely on the clinical diagnosis of PMR made by the attending physician. Although patients who were positive for ACPA or met the 2010 ACR/EULAR classification criteria for RA were excluded, the possibility remains that some patients with RA or those not meeting the PMR classification criteria were included. In addition, ESR data were missing in seven cases, ALP values in one case, RF values in four cases, ACPA values in 24 cases, and MMP-3 values in four cases. 4) Due to the stringent exclusion criteria applied to ensure a homogeneous study population, a considerable number of patients were excluded from the analysis. This rigorous selection process may have introduced selection bias, thereby limiting the generalizability of our findings, particularly to different ethnic populations and healthcare systems. 5) MTX was typically initiated late in the treatment course (median 151 days), often for patients with an inadequate response to glucocorticoids. This retrospective design means our findings cannot address the question of MTX's effectiveness as an early glucocorticoid-sparing agent.

## Conclusions

Although the addition of MTX did not significantly lower glucocorticoid dosage in the overall PMR population, our findings suggest a substantial benefit for patients with glucocorticoid-resistant PMR, markedly reducing cumulative glucocorticoid exposure and increasing the rate of discontinuation. Rather than uniformly introducing MTX for all PMR patients, it appears crucial to identify those who are likely to require intensified glucocorticoid therapy or who exhibit resistance. Clinicians may maximize MTX’s therapeutic potential and mitigate long-term glucocorticoid exposure by focusing on patient selection. Further large-scale, prospective studies are warranted to confirm these results and refine criteria for identifying patients who stand to benefit most from MTX in PMR management.
